# Poor mental health in Ghana: who is at risk?

**DOI:** 10.1186/1471-2458-13-288

**Published:** 2013-04-01

**Authors:** Heather Sipsma, Angela Ofori-Atta, Maureen Canavan, Isaac Osei-Akoto, Christopher Udry, Elizabeth H Bradley

**Affiliations:** 1 Department of Health Policy and Administration, Yale School of Public Health, New Haven, Connecticut, USA; 2 Department of Psychiatry, University of Ghana Medical School, Accra, Ghana; 3 Institute of Statistical, Social and Economic Research, University of Ghana, Legon, Ghana; 4 Department of Economics, Yale University, New Haven, Connecticut, USA

**Keywords:** Mental health, Ghana, Women, Empowerment

## Abstract

**Background:**

Poor mental health is a leading cause of disability worldwide with considerable negative impacts, particularly in low-income countries. Nevertheless, empirical evidence on its national prevalence in low-income countries, particularly in Africa, is limited. Additionally, researchers and policy makers are now calling for empirical investigations of the association between empowerment and poor mental health among women. We therefore sought to estimate the national prevalence of poor mental health in Ghana, explore its correlates on a national level, and examine associations between empowerment and poor mental health among women.

**Methods:**

We conducted a cross-sectional analysis using data from a nationally representative survey conducted in Ghana in 2009–2010. Interviews were conducted face-to-face with participants (N = 9,524 for overall sample; n = 3,007 for women in relationships). We used the Kessler Psychological Distress Scale (K10) to measure psychological distress and assessed women’s attitudes about their roles in decision-making, attitudes towards intimate partner violence, partner control, and partner abuse. We used weighted multivariable multinomial regression models to determine the factors independently associated with experiencing psychological distress for our overall sample and for women in relationships.

**Results:**

Overall, 18.7% of the sample reported either moderate (11.7%) or severe (7.0%) psychological distress. The prevalence of psychological distress was higher among women than men. Overall, the prevalence of psychological distress differed by gender, marital status, education, wealth, region, health and religion, but not by age or urban/rural location. Women who reported having experienced physical abuse, increased partner control, and who were more accepting of women’s disempowerment had greater likelihoods of psychological distress (P-values < 0.05).

**Conclusions:**

Psychological distress is substantial among both men and women in Ghana, with nearly 20% having moderate or severe psychological distress, an estimate higher than those found among South African (16%) or Australian (11%) adults. Women who are disempowered in the context of intimate relationships may be particularly vulnerable to psychological distress. Results identify populations to be targeted by interventions aiming to improve mental health.

## Background

Approximately one of every four people suffer from poor mental health, making it a leading cause of disability around the globe [1]. Poor mental health increases susceptibility to both infectious and chronic diseases and accounts for more than 30% of years of life lost worldwide [1,2]. Additionally, the negative economic impact of mental health issues is considerable [3], particularly in low-income countries where key risk factors including poverty, underemployment and unemployment, political instability, and HIV/AIDS are most prevalent.

Nevertheless, empirical evidence on national prevalence of poor mental health in low-income countries, particularly in Africa, is limited. Although several studies have been conducted in African countries, most have used small, specialized populations including people living in rural settings [4-6], pregnant women [7], and hospitalized patients [8,9] and thus lack national generalizability. Due in large part to the diversity of populations examined and methods used, prevalence estimates of poor mental health have varied widely, ranging from 4% to 65% [4-15]. The Nigerian Survey of Mental Health and Well-Being (NSMHW) is the only survey from the World Health Organization’s Mental Health Survey Initiative that was conducted in a low-income African country [16]. These data, however, are not nationally representative and were collected nearly a decade ago, limiting their usefulness for estimating contemporary estimates of poor mental health.

Although research that examines correlates of poor mental health within African countries are limited in number, findings consistently indicate that lower socioeconomic status, less education, and female gender are risk factors for poorer mental health [6-9,12,13,15]. Based on this evidence, researchers and policy makers are now calling for empirical investigations of the association between empowerment and poor mental health among women. Although this link has been suggested by a few small studies in Africa [7,11-13], a more thorough understanding of this association may provide avenues for interventions.

In this study, we sought to estimate the national prevalence of poor mental health in Ghana, a low-income country in West Africa, and to explore its correlates on a national level. Additionally, we aimed to examine associations between empowerment and poor mental health among women. Ghana is particularly relevant to this discourse as country leadership is poised to make substantial improvements in mental health services since the recent passing of their Mental Health Bill, which calls for better integration of mental health into the Ghanaian National Health Services (Mental Health Act 846 of 2012). Consequently, current epidemiological data on mental health and its correlates may be useful in understanding the scope of the problem in Ghana and for targeting particular subgroups for interventions.

## Methods

### Study design and sample

We conducted a cross-sectional analysis using data from a nationally representative survey conducted in Ghana in 2009–2010 by the Economic Growth Center (EGC) at Yale University and the Institute of Statistical, Social, and Economic Research (ISSER) at the University of Ghana, Legon. The data is not currently openly available; permission to use the data was granted by Christopher Udry, director of the EGC and Professor of Economics at Yale University. This survey is the first in a planned series, entitled the Ghana Socioeconomic Panel Survey, designed to provide a scientific framework for understanding change in the process of development in Ghana.

The survey used a two-stage stratified sample design in which enumeration areas (EA) throughout the 10 regions in Ghana were randomly selected, in proportion to 2009 regional population estimates, and then 15 households were randomly selected from each EA. EAs were oversampled in the Upper East and Upper West regions to allow for a sufficient number of households to be interviewed. A total of 5009 households from 334 EAs were interviewed. Less than one percent of households (32 households) refused to be interviewed.

Interviews were conducted face-to-face with participants. Seventeen interview teams each consisted of a supervisor, a senior interviewer, four interviewers, and a driver. Questions on mental health were individually asked of the head of each household, the first spouse, and one other household member older than age 12, selected at random. Our overall analytic sample included 9,524 participants. Questions on women’s empowerment were asked of women who reported either currently being in a relationship or having been in a relationship in the last 12 months. This subsample included 3,007 women.

### Measures

#### Outcome

We used the Kessler Psychological Distress Scale (K10) to measure psychological distress. The K10 has been used in several countries and has been validated in a low-income, African setting with strong validity and reliability [17-21]. This measure has also been shown to be associated with the Composite International Diagnostic Interview (CIDI) and has been used to indicate a high probability of meeting criteria for a DSM-IV mental disorder [17]. The K10 asks participants to respond to 10 questions about the frequency with which they have experienced specific feelings in the last four weeks. Responses ranged on a 5-point scale from “none of the time” (1) to “all of the time” (5). Questions included “About how often did you feel tired out for no good reason?” and “About how often did you feel hopeless?” Scores were summed across items for a possible minimum score of 10 and maximum score of 50, with lower scores indicating lower psychological distress. Scores were additionally grouped into four categories, using a conservative approach suggested by past literature [17] and which has been used in similar populations [20,21]: 10–19 (likely to be well), 20–24 (likely to have mild psychological distress), 25–29 (likely to have moderate psychological distress), and 30–50 (likely to have severe psychological distress). For analysis, we combined respondents likely to be well and likely to have mild distress to serve as our reference category.

#### Independent variables

We examined sociodemographic characteristics as possible correlates of psychological distress based on the literature [4,13,20]. Measures were self-reported and included age in years, marital status (never married, married, and formerly married), education (none, primary or less, middle, and secondary or above), general health status (very healthy, somewhat healthy, and unhealthy), religion (Christian, Muslim, traditional, and no religion), residential location (urban and rural), geographical region (10 regions in Ghana), and overall wealth. Wealth was based on a 5-level household asset index constructed with principal component analysis of 47 groupings of durable assets and living conditions (including ownership of a stove, refrigerator, computer or air conditioner and if the household uses safe roofing material or electricity for cooking or lighting) [22].

We measured women’s empowerment in several ways; measures were self-reported and were selected based on availability and use in prior literature [13,23]. First, we assessed women’s attitudes about their roles in decision-making with two items: “important decisions should be made only by the men in the family,” and “a wife has a right to express her opinion.” Participants indicated whether they agreed or disagreed with these statements. Second, we assessed attitudes towards intimate partner violence with one item, “a wife should tolerate being beaten in order to keep the family together,” with which participants indicated whether they agreed or disagreed. Third, we assessed partner control with four items (agree/disagree) based on their relationship in the last 12 months: 1) “partner frequently accused [name] of being unfaithful,” 2) “partner frequently tried to limit [name’s] contact with her family,” 3) “partner insisted on knowing where [name] was at all times,” and 4) “partner did not trust [name] with money.” Partner control was scored by tallying the number of items with which the women agreed and thus ranged from 0 to 4. Higher scores indicated greater partner control among women. Last, participants indicated whether or not they had been emotionally abused (including being insulted or threatened) and physically abused (including being pushed, hit, slapped, having had something thrown at them, being kicked, dragged or beaten up) in the last 12 months by their partners.

### Statistical analysis

We first generated weighted frequencies to describe the overall levels of psychological distress experienced by our sample participants and then compared levels of psychological distress by gender using a Rao-Scott chi-square test. We then generated weighted frequencies and means to describe the sociodemographic characteristics of our sample and levels of empowerment experienced by women in relationships. We compared these characteristics by level of psychological distress using weighted regression models and Rao-Scott chi-square tests, for continuous and categorical variables, respectively. We constructed weighted multivariable multinomial regression models to determine the factors that were independently associated with experiencing moderate and severe psychological distress for the sample overall and for the sample of women in relationships. We also tested whether the associations between independent variables and psychological distress differed significantly by gender, because differences by gender for risk for poor mental health have been consistently documented in the literature [6-9,12,13,15]. We tested gender as an effect modifier in our overall sample by including interaction terms of each potential covariate and gender one at a time in our final multivariable model. All analyses accounted for the complex sampling design of the survey. We conducted a complete case analysis because missing data was minimal (<5.5%). SAS 9.2 (Carey, NC) was used for all analyses.

## Results

### Sample characteristics

Participants were 38 years old on average, 56% were female, and 63% were married (Table 1). Approximately 30% had no education and 13% had a secondary education or higher. One-third of participants lived in an urban area, and most participants reported being “very healthy.” The sample was distributed throughout the 10 regions of Ghana in proportion to their regional populations. Approximately 72% were Christian, 18% were Muslim, 6% reported believing in a traditional religion, and 5% reported having no religion. Among women in relationships in the last year (n = 3,007), 87% believed a wife has the right to express her opinion, but 30% believed important decisions should be made only by the men of the family. One in four women also believed a wife should tolerate being beaten in order to keep the family together. Approximately 27% had experienced emotional abuse, and 5% had experienced physical abuse in the past 12 months by their partner.

**Table 1:** **Participant characteristics over all and by psychological distress status (column%)**^**a**^

	**Overall** **(N = 9,524)**	**Well/Mild** **(81.3%)**	**Moderate** **(11.7%)**	**Severe** **(7.0%)**
Age	37.8 (0.20)	36.5 (0.22)	42.4 (0.62)	45.8 (0.80)
Gender				
Male	44.0%	45.8%	34.8%	38.8%
Female	56.0%	54.2%	65.2%	61.2%
Marital status				
Married	62.7%	60.7%	72.6%	69.4%
Formerly married	10.0%	8.7%	14.3%	17.9%
Never married	27.3%	30.6%	13.1%	12.7%
Education				
None	30.4%	25.3%	49.0%	58.0%
Primary or less	20.6%	21.6%	16.7%	14.8%
Middle/JSS	36.5%	39.1%	26.5%	22.3%
Secondary and above	12.6%	14.0%	7.9%	4.9%
Residential location				
Urban	33.6%	35.7%	24.5%	23.8%
Rural	66.4%	64.3%	75.5%	76.2%
Region				
Western	10.0%	10.1%	11.5%	7.2%
Central	9.5%	9.9%	7.6%	7.4%
Gt. Accra	10.5%	11.8%	6.1%	2.8%
Volta	10.4%	10.7%	10.3%	7.0%
Eastern	11.3%	11.0%	11.4%	14.4%
Ashanti	18.1%	19.3%	11.8%	14.4%
Brong Ahafo	7.7%	7.9%	7.2%	5.8%
Northern	12.6%	10.0%	19.9%	30.7%
Upper East	5.5%	4.9%	9.5%	6.7%
Upper West	4.4%	4.4%	4.8%	3.6%
Wealth index				
First quintile (Poorest)	19.9%	19.3%	24.0%	20.0%
Second quintile	20.7%	19.0%	26.6%	31.1%
Third quintile	18.4%	19.3%	15.1%	13.9%
Fourth quintile	19.2%	19.1%	18.8%	21.5%
Fifth quintile (Wealthiest)	21.7%	23.3%	15.6%	13.5%
Self-rated health				
Very healthy	77.2%	80.9%	63.2%	57.8%
Somewhat healthy	15.9%	14.0%	22.9%	26.3%
Unhealthy	6.9%	5.1%	13.9%	15.9%
Religion				
Christian	71.9%	74.9%	61.5%	54.6%
Muslim	17.5%	16.1%	21.4%	27.2%
Traditional	5.8%	4.7%	10.6%	10.8%
No religion	4.8%	4.3%	6.6%	7.3%
*Women who have been in a relationship in the past 12 months*				
Important decisions should be made only by the men of the family				
Agree	30.4%	27.6%	39.6%	42.3%
Disagree	69.6%	72.4%	60.4%	57.7%
A wife has a right to express her opinion				
Agree	86.5%	87.6%	82.9%	81.9%
Disagree	13.5%	12.4%	17.1%	18.1%
A wife should tolerate being beaten in order to keep the family together				
Agree	23.6%	19.7%	35.1%	42.6%
Disagree	76.4%	80.3%	64.9%	57.4%
Partner control^+^ (Range: 0 – 4)	0.5 (0.02)	0.5 (0.02)	0.8 (0.04)	0.9 (0.06)
Emotional abuse				
Yes	26.8%	25.5%	33.0%	27.8%
No	73.2%	74.5%	67.0%	72.2%
Physical abuse				
Yes	4.6%	4.0%	5.1%	9.5%
No	95.4%	96.0%	94.9%	90.5%

^a^Values presented are Mean (SE) and valid column% for continuous and categorical variables, respectively.

^+^Partner control is the number of the following statements to which participants responded “yes”: 1) Partner frequently accused [name] of being unfaithful; 2) Partner frequently tried to limit [name’s] contact with her family; 3) Partner insisted on knowing where [name] was at all times; 4) Partner did not trust [name] with money.

Overall, 18.7% of the sample reported moderate (11.7%) or severe (7.0%) psychological distress. The prevalence of psychological distress was higher among women than men; 21.2% of women reported moderate (13.6%) or severe (7.6%) psychological distress compared with 15.5% of men reported either moderate (9.3%) or severe (6.2%) psychological distress.

### Factors associated with psychological distress

In the bivariate analysis, age, gender, marital status, education, residential location, region, wealth, self-rated health, and religion were significantly associated with having psychological distress (P-values < 0.01). Our multivariable model indicated that females had 43% greater odds of moderate psychological distress than males (OR = 1.43; 95% CI = 1.23, 1.67), but were not significantly different from males in odds of severe psychological distress (OR = 1.16; 95% CI = 0.95, 1.41; Table 2). Compared with participants who had never been married, married participants had twice the odds of moderate but not severe psychological distress (OR = 1.95; 95% CI = 1.48, 2.57 and OR = 1.29; 95% CI = 0.88, 1.89, respectively), whereas formerly married participants (e.g., separated/divorced, widowed) had double the odds of both moderate and severe psychological distress (OR = 2.23; 95% CI = 1.48, 3.35 and OR = 1.98; 95% CI = 1.20, 3.27, respectively). Participants with no education had significantly higher odds of moderate and severe distress (OR = 1.67; 95% CI = 1.17, 2.39 and OR = 2.92; 95% CI = 1.82, 4.69, respectively), and participants with primary or less education had significantly greater odds of severe distress (OR = 1.69; 95% CI = 1.05, 2.71) than participants with secondary or more education. Participants in the second wealth quintile had significantly greater odds of both moderate and severe psychological distress than participants in the wealthiest (fifth) wealth quintile (OR = 1.83; 95% CI = 1.23, 2.74 and OR = 2.10; 95% CI = 1.34, 3.28, respectively). Similarly, participants in the fourth wealth quintile had greater odds of moderate psychological distress than participants in the wealthiest quintile (OR = 1.53; 95% CI = 1.06, 2.19). Participants living in all regions except the Upper West and Central regions of Ghana had higher odds of psychological distress than those living in Greater Accra. Additionally, participants with worse self-rated health had greater odds of moderate and severe psychological distress. Last, participants who reported having no religion had greater odds of moderate but not severe psychological distress compared with participants who reported being Christian (OR = 1.50; 95% CI = 1.02, 2.19).

**Table 2:** Multinomial regression examining the relationship between psychological distress and sociodemographic characteristics; overall sample (N = 9,099)

	**OR (95% CI)**		
	**Well/Mild**	**Moderate**	**Severe**
Age	1.00	1.00 (0.99, 1.01)	1.01 (1.00, 1.02)
Gender			
Male	1.00	1.00	1.00
Female	1.00	1.43 (1.23, 1.67)**	1.16 (0.95, 1.41)
Marital status			
Never married	1.00	1.00	1.00
Married	1.00	1.95 (1.48, 2.57)**	1.29 (0.88, 1.89)
Formerly married	1.00	2.23 (1.48, 3.35)**	1.98 (1.20, 3.27)**
Education			
None	1.00	1.67 (1.17, 2.39)**	2.92 (1.82, 4.69)**
Primary or less	1.00	1.19 (0.82, 1.72)	1.69 (1.05, 2.71)*
Middle/JSS	1.00	1.00 (0.72, 1.40)	1.34 (0.87, 2.08)
Secondary and above	1.00	1.00	1.00
Residential location			
Urban	1.00	0.85 (0.67, 1.09)	1.03 (0.78, 1.37)
Rural	1.00	1.00	1.00
Wealth index			
First quintile	1.00	1.07 (0.71, 1.59)	0.96 (0.59, 1.57)
Second	1.00	1.83 (1.23, 2.74)**	2.10 (1.34, 3.28)**
Third	1.00	1.09 (0.73, 1.62)	0.96 (0.57, 1.61)
Fourth	1.00	1.53 (1.06, 2.19)*	1.52 (0.98, 2.36)
Fifth	1.00	1.00	1.00
Region			
Western	1.00	1.80 (1.10, 2.96)*	2.53 (1.28, 5.01)**
Central	1.00	0.85 (0.50, 1.44)	1.65 (0.84, 3.25)
Gt. Accra	1.00	1.00	1.00
Volta	1.00	1.58 (0.97, 2.59)	2.39 (1.20, 4.78)*
Eastern	1.00	1.72 (1.08, 2.73)*	5.03 (2.73, 9.26)**
Ashanti	1.00	1.10 (0.68, 1.77)	3.00 (1.60, 5.62)**
Brong Ahafo	1.00	1.59 (0.94, 2.68)	3.03 (1.46, 6.28)**
Northern	1.00	2.05 (1.22, 3.45)**	6.51 (3.46, 12.24)**
Upper East	1.00	2.67 (1.52, 4.69)**	4.31 (2.07, 8.98)**
Upper West	1.00	1.29 (0.71, 2.36)	2.13 (0.98, 4.64)
Self-rated health			
Very healthy	1.00	1.00	1.00
Somewhat healthy	1.00	1.75 (1.40, 2.19)**	1.92 (1.45, 2.55)**
Unhealthy	1.00	2.81 (2.12, 3.72)**	2.98 (2.18, 4.09)**
Religion			
Christian	1.00	1.00	1.00
Muslim	1.00	1.03 (0.75, 1.40)	1.10 (0.75, 1.60)
Traditional	1.00	1.37 (0.96, 1.96)	1.22 (0.79, 1.89)
No religion	1.00	1.50 (1.02, 2.19)*	1.51 (0.97, 2.33)

**p < 0.01; *p ≤ 0.05.

### Factors associated with psychological distress: differences by gender

Interactions with gender indicated that the associations between psychological distress and the risk factors of traditional religious practices and of wealth were significantly different for men than for women (P-values for interactions < 0.05). Among males, having traditional religious beliefs was associated with significantly greater odds of severe psychological distress compared with males who reported being Christian (OR = 1.83; 95% CI = 1.09, 3.09), whereas among females, having traditional religious beliefs was not associated with psychological distress (OR = 0.87; 95% CI = 0.53, 1.52). Similarly, among men, being less wealthy (2nd versus 5th quintile) was associated with 3 times the odds of severe psychological distress whereas among women, the effect of being less wealthy on psychological distress was associated with only 1.6 times the odds of severe psychological distress (95% CI = 1.00, 2.64).

### Factors associated with psychological distress among women in relationships

Among women who were in relationships in the last year, bivariate analyses indicated that low women’s empowerment, higher partner control, and experiences of abuse were all significantly associated with reporting psychological distress (P-values < 0.01; Table 1). In the multivariable analysis (Table 3), after adjusting for all other sociodemographic characteristics, women who reported having experienced physical abuse in the last 12 months had more than double the odds of severe psychological distress (OR = 2.22; 95% CI = 1.10, 4.49). Additionally, every additional circumstance of partner control (out of 4 possible circumstances) was associated with 55% and 82% greater odds of moderate and severe psychological distress, respectively (OR = 1.55; 95% CI = 1.28, 1.87 and OR = 1.82; 95% CI = 1.43, 2.32, respectively). Women who believed that a wife does not have a right to express her opinion had greater odds of moderate psychological distress (OR = 1.79; 95% CI = 1.24, 2.58), and women who agreed that wife should tolerate being beaten to keep the family together had greater odds of moderate and severe psychological distress (OR = 1.63; 95% CI = 1.18, 2.25 and OR = 2.61; 95% CI = 1.80, 3.78, respectively).

**Table 3:** Multinomial regression examining the relationship between psychological distress and sociodemographic and empowerment characteristics; women in relationships (N = 2,849)

	**OR (95% CI)**		
	**Well/Mild**	**Moderate**	**Severe**
Age	1.00	1.00 (0.99, 1.01)	1.01 (0.99, 1.02)
Marital status			
Never married	1.00	1.00	1.00
Married	1.00	0.87 (0.47, 1.63)	0.50 (0.22, 1.17)
Formerly married	1.00	1.24 (0.43, 3.59)	0.84 (0.26, 2.76)
Education			
None	1.00	2.05 (1.06, 3.94)*	3.39 (1.17, 9.81)*
Primary or less	1.00	1.54 (0.77, 3.09)	2.12 (0.71, 6.34)
Middle/JSS	1.00	1.31 (0.71, 2.43)	1.20 (0.41, 3.50)
Secondary and above	1.00	1.00	1.00
Residential location			
Urban	1.00	1.21 (0.85, 1.72)	1.60 (1.06, 2.39)*
Rural	1.00	1.00	1.00
Wealth index			
First quintile	1.00	1.77 (1.01, 3.11)*	1.33 (0.62, 2.86)
Second	1.00	2.13 (1.26, 3.60)**	1.59 (0.76, 3.31)
Third	1.00	1.22 (0.68, 2.20)	1.33 (0.55, 3.23)
Fourth	1.00	1.95 (1.14, 3.34)*	1.54 (0.74, 3.20)
Fifth	1.00	1.00	1.00
Region			
Western	1.00	1.54 (0.72, 3.28)	2.67 (0.66, 10.89)
Central	1.00	0.46 (0.20, 1.06)	2.08 (0.52, 8.41)
Gt. Accra	1.00	1.00	1.00
Volta	1.00	1.31 (0.63, 2.73)	1.93 (0.46, 8.21)
Eastern	1.00	1.41 (0.66, 3.00)	6.09 (1.60, 23.24)**
Ashanti	1.00	0.45 (0.18, 1.12)	2.06 (0.49, 8.69)
Brong Ahafo	1.00	1.06 (0.43, 2.64)	1.89 (0.38, 9.29)
Northern	1.00	1.29 (0.58, 2.89)	2.78 (0.71, 10.87)
Upper East	1.00	1.55 (0.65, 3.73)	2.62 (0.62, 11.06)
Upper West	1.00	0.71 (0.28, 1.79)	0.75 (0.15, 3.67)
Self-rated health			
Very healthy	1.00	1.00	1.00
Somewhat healthy	1.00	1.54 (1.10, 2.16)*	2.57 (1.65, 4.00)**
Unhealthy	1.00	2.38 (1.51, 3.73)**	2.10 (1.22, 3.62)**
Religion			
Christian	1.00	1.00	1.00
Muslim	1.00	0.82 (0.53, 1.26)	1.04 (0.56, 1.94)
Traditional	1.00	1.16 (0.71, 1.90)	0.79 (0.40, 1.58)
No religion	1.00	1.33 (0.74, 2.40)	1.29 (0.64, 2.62)
Important decisions should be made only by the men in the family	1.00	1.39 (0.99, 1.94)	1.48 (0.96, 2.28)
A wife does not have a right to express her opinion	1.00	1.79 (1.24, 2.58)**	1.68 (0.96, 2.93)
A wife should tolerate being beaten in order to keep the family together	1.00	1.63 (1.18, 2.25)**	2.61 (1.80, 3.78)**
Partner control (0–4)	1.00	1.55 (1.28, 1.87)**	1.82 (1.43, 2.32)**
Emotional abuse	1.00	1.20 (0.88, 1.63)	0.77 (0.49, 1.20)
Physical abuse	1.00	0.88 (0.45, 1.74)	2.22 (1.10, 4.49)*

**p < 0.01; *p ≤ 0.05.

## Discussion

Our findings indicate that the prevalence of reported psychological distress is substantial among both men and women in Ghana, but is higher among women. Nearly 20% of the sample had moderate or severe psychological distress, an estimate higher than that found among South African (16%) [20] and Australian (11%) adults [24]. Our findings are consistent with literature supporting women’s vulnerability to poor mental health [14,25], but this is the first study to our knowledge that has been conducted among a large, nationally representative sample in a low-income country on the African continent. Our results also suggest that women who are disempowered and lack control in the context of intimate relationships may be particularly vulnerable to psychological distress. Although small studies from several African countries have suggested an association between empowerment and mental health [10-13], our study is the first to explore multiple measures of empowerment, using a nationally representative sample of women.

Overall, the prevalence of psychological distress differed by gender, marital status, education, wealth, region, health and religion, but not by age or urban/rural location. These results are consistent with other studies that demonstrate higher rates of poor mental health among women and those with lower education [12,13]. The literature, however, is less consistent on the associations between marital status and poor mental health [4-7,9]. In high income countries, for instance, females who have never been married often demonstrate greater risk for poor mental health than females who are married [26]. In low income countries, however, this association may be reversed. Given the dependence of women on men in these settings for income, we hypothesize that marriage can bring with it substantial disempowerment and lack of control, which we found to increase risk for psychological distress. As a result, in these contexts, never having been married may in fact be protective [4,6,10,11,25]. Future research should explore measures of empowerment among women who have never been married to more clearly understand the mechanisms linking mental health and marital status.Our analysis of women in relationships suggests that empowerment is particularly important for women’s psychological health in Ghana and possibly in other low-income African countries. All of the components of women’s empowerment, including attitudes about their roles in decision-making, attitudes towards intimate partner violence, partner control, and experiences of abuse, were strongly associated with worse mental health and present opportunities for intervention. Interventions may focus on preventing experiences of intimate partner violence among women and decreasing the acceptance of such behavior [27,28]. They may also aim to decrease the effects of intimate partner violence on women’s mental health by ensuring and building adequate social support for her if physical and/or psychological abuse persists [29,30]. Interventions may also aim to target men in an effort to reshape their views on physical and psychological abuse [31]. More intervention studies, however, are needed in low income settings; our research suggests that interventions targeting multiple components of empowerment will have potentially greater effects than those targeting fewer aspects of women’s empowerment.

Our findings have implications for policy at the national level for Ghana and possibly for other low-income African countries as well. First, given the high prevalence of psychological distress among the population, a greater portion of national healthcare budgets should be allocated to mental health. Second, because poor mental health is associated with several negative outcomes, including a reduced ability to pursue educational and employment opportunities, efforts to improve access to treatment and reduce the stigma associated with poor mental health are essential. Fundamental to addressing the high need for mental health services is integrating mental health care into primary care. This calls for approaches such as task shifting and expanded community-based mental health. Prioritizing mental health services and rehabilitation is particularly relevant in Ghana, where a new mental health bill has been passed and where they are currently developing approaches to deploy university graduates in psychology to fulfill their national service obligation as community mental health workers. This with the expansion of brief assessment units in the psychiatric hospitals and new psychiatric wings in regional hospitals are planned to strengthen access to needed services.

Although our study has several strengths, including the use of nationally representative data, it does have limitations. All data were self-reported and due to resource constraints could not be corroborated by clinical interviews or additional sources; nevertheless, this method is the standard approach for measuring mental health and sociodemographic characteristics in population-based surveys and was used consistently across the sample. Wealth is particularly difficult to measure in Ghana as much is generated from the informal sector. Furthermore, our measure of mental health does not indicate whether or not the respondent has a clinically significant impairment. Previous literature [17], however, suggests a high correlation between the K10 and clinical mental health disorders (anxiety, affective, substance use, or personality disorders); among people with a K10 of 26 or above, at least 75% can be expected to have a clinically significant mental disorder. There still exists, however, the potential for misclassification. Additionally, our analysis examining empowerment only studies women who have been involved in romantic relationships in the past 12 months. Future research should improve methodology and measures to examine this construct among women outside of romantic relationships. Last, the cross-sectional nature of the analysis limits our ability to understand the temporal sequence between mental health and empowerment. The study does, however, identify a population who is suffering and should be targeted by interventions. It also highlights the importance of gender in low income countries as a critical public health issue.

## Conclusion

The prevalence of psychological distress is high among both men and women in Ghana. Women who are disempowered in the context of intimate relationships may be particularly vulnerable to psychological distress. This is the first study conducted among a large, nationally representative sample in a low-income country on the African continent, and our results suggest that findings from other contexts may not be generalizable to lower income African populations. Future studies should continue exploring risk factors for poor mental health among low-income African countries, and prevention and treatment should be delivered accordingly. Mental health services should be prioritized at the national level.

## Competing interests

The authors declared that they have no competing interest.

## Authors’ contributions

CU and IOA conceptualized and helped conduct the national study and provided comments on the final version of the manuscript. HS, AOA, MC, and EB conceptualized the analysis and interpreted results. HS conducted the analysis and drafted the paper. All authors critically reviewed and edited the paper and approve its final version.

## Pre-publication history

The pre-publication history for this paper can be accessed here:

http://www.biomedcentral.com/1471-2458/13/288/prepub
